# THE DIGITAL DIVIDE AMONG CHINESE OLDER ADULTS: INTERNET USAGE AND EHEALTH LITERACY

**DOI:** 10.1093/geroni/igae098.3740

**Published:** 2024-12-31

**Authors:** Jiaan Zhang

**Affiliations:** Fudan University, Fudan University, Shanghai, China (People’s Republic)

## Abstract

In recent years, particularly due to the COVID-19 pandemic, digital dependency has grown significantly in China, exacerbating the digital divide as a pressing social issue. This study examines Internet usage and eHealth literacy among Chinese older adults aged 60 and above compared to their younger counterparts, using data from the newly available 2021 China General Social Survey. Internet usage was assessed by asking respondents whether they currently use the Internet. eHealth literacy was measured using the 8-item eHealth Literacy Scale, with each item scored on a 5-point Likert scale. Age groups were compared using chi-square tests and t-tests. Correlates of Internet usage and eHealth literacy were analyzed with logistic regression and OLS regression models, respectively. The results indicate that older adults significantly lag in Internet usage, with only 35.25% of those aged 60 and above using the Internet, compared to 89.09% of the under-60 group. Additionally, the younger group demonstrated significantly higher eHealth literacy scores. Regression analyses further revealed that the negative association between older age and Internet usage is more pronounced among rural residents, and that older age is more strongly associated with lower eHealth literacy scores in both women and rural residents, controlling for sociodemographic and socioeconomic status, social support, and health status. This study provides the most recent evidence of significant disparities between older adults and other age groups in China regarding Internet usage and eHealth literacy. It underscores the need for policies and programs focused on bridging the digital divide among older adults.

